# Development and validation of a novel immune-related prognostic model in hepatocellular carcinoma

**DOI:** 10.1186/s12967-020-02255-6

**Published:** 2020-02-11

**Authors:** Zheng Wang, Jie Zhu, Yongjuan Liu, Changhong Liu, Wenqi Wang, Fengzhe Chen, Lixian Ma

**Affiliations:** 1 Department of Infectious Diseases, Qilu Hospital, Shandong University, Wenhua Xi Road 107, Jinan, 250012 Shandong China; 2 Department of Gastroenterology, Shandong Provincial Qianfoshan Hospital, The First Hospital Affiliated With Shandong First Medical University, Jingshi Road 16766, Jinan, 250014 Shandong China; 3 Shandong Center for Disease Control and Prevention, Health Education Institute, Jinan, 250000 Shandong China

**Keywords:** Hepatocellular carcinoma, Immune related gene, Prognosis, Prognostic signature, Bioinformatics

## Abstract

**Background:**

Growing evidence has suggested that immune-related genes play crucial roles in the development and progression of hepatocellular carcinoma (HCC). Nevertheless, the utility of immune-related genes for evaluating the prognosis of HCC patients are still lacking. The study aimed to explore gene signatures and prognostic values of immune-related genes in HCC.

**Methods:**

We comprehensively integrated gene expression data acquired from 374 HCC and 50 normal tissues in The Cancer Genome Atlas (TCGA). Differentially expressed genes (DEGs) analysis and univariate Cox regression analysis were performed to identify DEGs that related to overall survival. An immune prognostic model was constructed using the Lasso and multivariate Cox regression analyses. Furthermore, Cox regression analysis was applied to identify independent prognostic factors in HCC. The correlation analysis between immune-related signature and immune cells infiltration were also investigated. Finally, the signature was validated in an external independent dataset.

**Results:**

A total of 329 differentially expressed immune‐related genes were detected. 64 immune‐related genes were identified to be markedly related to overall survival in HCC patients using univariate Cox regression analysis. Then we established a TF-mediated network for exploring the regulatory mechanisms of these genes. Lasso and multivariate Cox regression analyses were applied to construct the immune-based prognostic model, which consisted of nine immune‐related genes. Further analysis indicated that this immune-related prognostic model could be an independent prognostic indicator after adjusting to other clinical factors. The relationships between the risk score model and immune cell infiltration suggested that the nine-gene signature could reflect the status of tumor immune microenvironment. The prognostic value of this nine-gene prognostic model was further successfully validated in an independent database.

**Conclusions:**

Together, our study screened potential prognostic immune-related genes and established a novel immune-based prognostic model of HCC, which not only provides new potential prognostic biomarkers and therapeutic targets, but also deepens our understanding of tumor immune microenvironment status and lays a theoretical foundation for immunotherapy.

## Background

Globally, liver cancer is known as the sixth leading cancer, and has the second-highest number of deaths [1]. All over the world, more than 600,000 people die of liver cancer each year, and nearly 850,000 new cases occur [1, 2]. Hepatocellular carcinoma (HCC) accounts for 85–90% of all liver cancers and has received public attention. Despite rapid advances in new tests and treatments, the 5-year survival rate for HCC is still less than one in five [3]. At present, surgery is still the main treatment for early liver cancer [4]. However, a significant proportion of patients will have postoperative recurrence or distant metastasis [5]. Recently, drugs such as sorafenib and regorafenib have been shown to be effective against advanced HCC [6, 7]. It is worth mentioning that patients with the same pathological type and clinical stage often have different outcomes after the same treatment, which is mainly due to the genetic heterogeneity of patients [8].

The immune system is thought to be a decisive factor in the development of cancer [9, 10], including HCC. Immune cells are major components of the tumor microenvironment and play a role in many key steps of HCC development from tumor growth to the development of metastasis [11, 12]. Besides, a large amount of inflammatory mediators were found to be associated with HCC development. IL-22, belongs to the cytokine family, was overexpressed in the HCC microenvironment and leading to tumor growth [13]. Chemokines (e.g. CXCL12, CCL20), as the immune molecules members of immune system, also play a crucial role in HCC growth, invasion and metastasis [14, 15]. It is proven that immunogenicity makes the immunotherapy of HCC a promising prospect [16]. Research progress discovery that programmed cell death-1 (PD-1) pathway is a new target for HCC immunotherapy [17]. As an anti-PD-1 monoclonal antibody, nivolumab can block PD-1 and restore the body’s anticancer immune response by interfering with the signaling pathway, thereby preventing T cell activation [18]. In HCC, nivolumab showed significant benefits in objective response rates and overall survival [19]. Therefore, nivolumab may provide a safe, effective and promising treatment for HCC [20]. Increasing studies have suggested that immune-related genes in HCC are closely related to the tumorigenesis and development of HCC [21]. However, there is currently no prognostic model based on immune-related genes to systematically evaluate tumor immune environment and predict the overall prognosis of HCC patients. Therefore, the construction of an immune-based prognosis model that can reliably predict HCC prognosis is of great clinical significance.

In the first step of this study, we screened differentially expressed immune-related genes closely related to HCC through bioinformatics analysis of large-scale sequencing database. Next, immune‐related genes significantly related to prognosis were further detected. Then we constructed an immune-related prognostic model by integrating immune-related genes for HCC. Moreover, the prognostic value of our immune-related prognostic model was further validated in an independent International Cancer Genome Consortium (ICGC) database. We here aimed to provide novel biomarkers that would be effective in predicting the prognosis and monitoring tumor immune microenvironment in HCC patients.

## Methods

### Data collection

Gene expression data and clinical information of HCC samples were acquired from TCGA data portal (https://portal.gdc.cancer.gov/cart; up to September 16, 2019). Processed RNA-Seq FPKM data of 374 HCC and 50 adjacent normal HCC tissues were downloaded for further analyses. After careful search and examination, 224 HCC patients were accompanied by hepatitis B virus. This included 81 HBsAg positive patients, 60 HBsAg and HBsAb both positive patients, and 83 patients whose history risk factors were hepatitis B. The International Cancer Genome Consortium (ICGC; https://dcc.icgc.org/search?filters=%7B%22donor%22:%7B%22projectId%22:%7B%22is%22:%5B%22LIRI-JP%22%5D%7D,%22availableDataTypes%22:%7B%22is%22:%5B%22exp_seq%22%5D%7D%7D%7D) was a web-based portal that provided comprehensive molecular genetic profiles of 50 different tumor types. ICGC represents a valuable database for analyzing cancer genome at the genomic and transcriptomic levels. For validation cohort, gene expression data and the corresponding survival information of 231 HCC patients were retrieved from the ICGC database. We download 1811 immune-related genes via the Immunology Database and Analysis Portal (ImmPort; https://www.immport.org/shared/genelists) database, which contains 17 immune categories based on various molecular function [22]. The cistrome Cancer (http://cistrome.org/CistromeCancer/CancerTarget/) represents a useful database for biomedical and genetic research and includes totally 318 transcription factors (TFs) [23]. In order to investigate the regulatory mechanism of immune-related genes, we extracted these TFs for subsequent research. Because our data were downloaded directly from public databases and we strictly abided by the publishing guidelines provided by TCGA and ICGC, there were no requirement for ethical approvals.

### Differential expression analyses

The differentially expressed immune‐related genes and TFs in HCC and normal tissues were detected using the Wilcoxon test method in R. |log2 foldchange| > 1 and FDR < 0.05 were considered as significant. Heatmaps were generated using pheatmap package and volcano plots were also conducted in R software. To assess the potential biologic functions of differentially expressed immune‐related genes, Gene Ontology (GO) [24] and Kyoto Encyclopedia of Genes and Genomes (KEGG) pathway enrichment analysis [25] were performed by the cluster Profiler package [26] in R. Functional categories with a adjusted P value < 0.05 were considered as significant pathways.

### Survival analysis

Only patients with a follow-up time less than 2000 days were used for the survival analyses. To investigate the prognostic value of differentially expressed immune‐related genes in HCC patients, univariate Cox analysis was implemented by the survival package. Only these genes with a P value < 0.01 were considered as prognostic immune‐related genes. These prognostic immune‐related genes were further analyzed by GO and KEGG analysis. To evaluate how TFs regulating these immune‐related genes, we first screened prognosis-related TFs using univariate Cox analysis with a P value < 0.01 Then the correlation test between prognosis-related TFs and prognostic immune‐related genes was investigated. This step was performed using cor.test function in R, whose core method was Pearson test. The correlation coefficient and P value were calculated by cor.test. The cut-off criteria were set as correlation coefficient > 0.5 and P < 0.05. In order to make the picture clear, we only chose TFs that regulated more than nine immune‐related genes. Cytoscape was utilized for constructing and visualizing the regulatory network [27].

### Construction of the immune-related signature for HCC

To develop a prognostic model, Lasso and multivariate Cox regression analyses were utilized to assess the relationship between prognostic immune‐related genes expressions and overall survival (OS). To avoid over-fitting and delete highly related genes, Lasso Cox regression was carried out using survival and glmnet package. Genes detected via Lasso algorithm were evaluated by step wise multivariate Cox regression analysis. Risk scores were acquired based on genes expression multiplied a linear combination of regression coefficient obtained from the multivariate Cox regression. Patients were assigned to high risk and low risk groups according to the median risk score. The Kaplan–Meier analysis was performed to compare overall survival between high risk and low risk groups via survival package in R. The receiver operating characteristic (ROC) curve was implemented by the R software package survival ROC. In addition, univariate and multivariate analyses were utilized to assess the effect of risk scores on overall survival and several clinical features.

### Correlation analysis between immune-related signature and immune cells infiltration

To explore the associations between prognostic model and immune cells infiltration, we employed Tumor Immune Estimation Resource (TIMER) [28], a useful resource for comprehensive analysis of tumor-infiltrating immune cells. TIMER algorithm allows users to estimate the composition of six tumor-infiltrating immune cells subsets (B cells, CD4+ T cells, CD8+ T cells, macrophages, neutrophils, and dendritic cells). The immune infiltrate levels of HCC patients were derived from TIMER website and the correlation between the prognostic model and six tumor-infiltrating immune cells were conducted in R.

### Genetical alteration of the immune-related signature

The cBio Cancer Genomics Portal (CBioPortal) represents an important online platform for visualization and analysis of various cancer genomics data [29, 30]. CBioPortal was conducted to analyse genetic alterations of prognostic genes in HCC patients (TCGA, Provisional). Anti-cancer drugs that target these genes were also identified.

### External validation of the immune-related signature

To verify the prognostic value of immune-related signature risk score model, we used the ICGC database as the validation cohort. The same formula was used to calculate risk scores and patients were classified into high risk and low risk groups based on the optimal cut-off point. Kaplan–Meier and ROC curve analyses were carried out as described above.

### Statistical analysis

All analyses were performed using Rversion 3.5.1. Unless otherwise noted, P < 0.05 was considered to be significant.

## Results

### Differentially expressed immune‐related genes and TFs in HCC

**Fig. 1:**
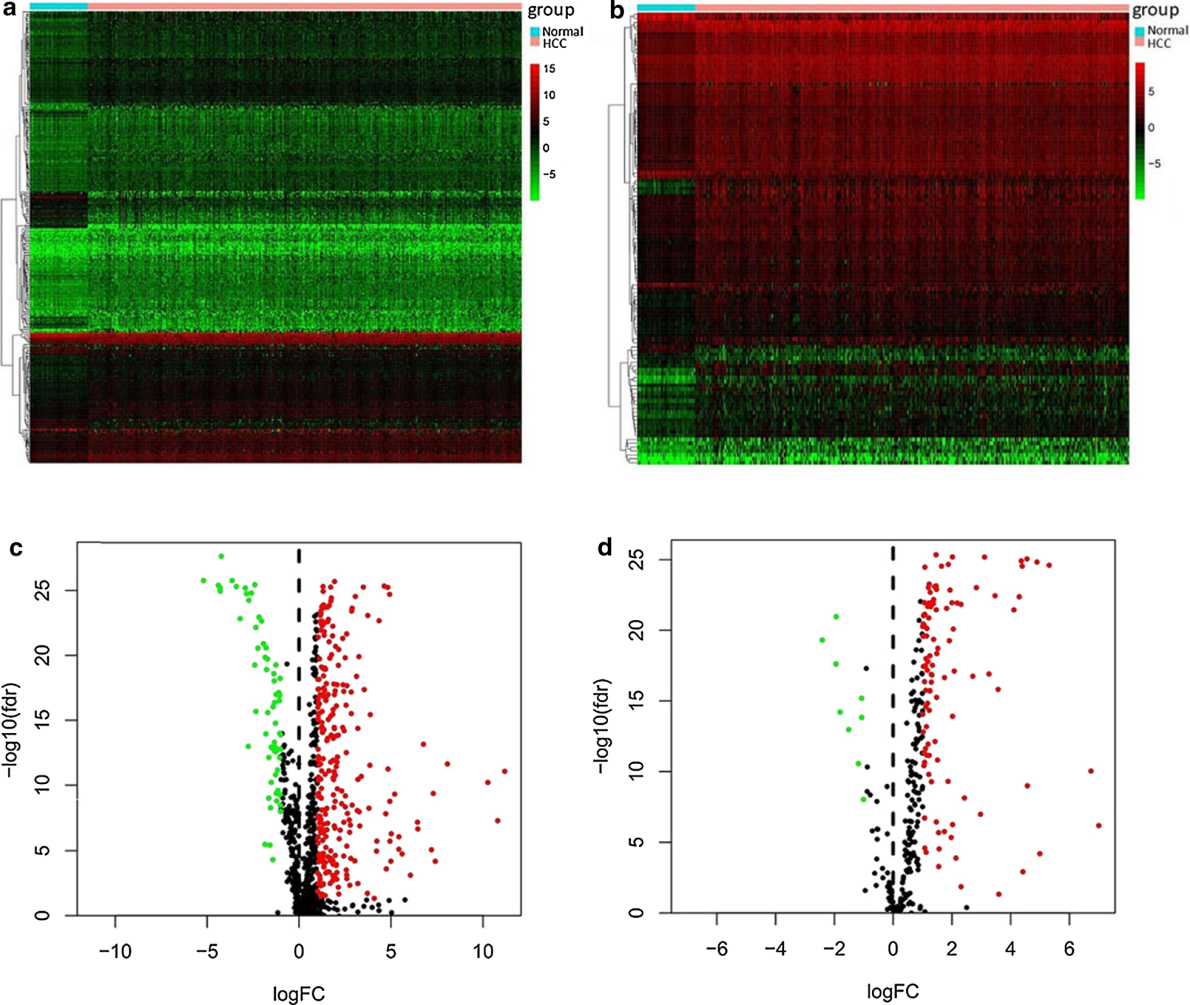
Differentially expressed immune-related genes and transcription factors (TFs) in hepatocellular carcinoma (HCC). **a** Heatmap of significantly differentially expressed immune-related genes in HCC. The color from green to red represents the progression from low expression to high expression. **b** Volcano plot of differentially expressed immune-related genes. The red dots in the plot represents upregulated genes and green dots represents downregulated genes with statistical significance. Black dots represent no differentially expressed genes. **c** Heatmap of significantly differentially expressed TFs in HCC. Red represents higher expression while green represents lower expression. **d** Volcano plot of differentially expressed TFs in HCC. Colored dots represent differentially expressed TFs and black dots represent no differentially expressed TFs

**Fig. 2:**
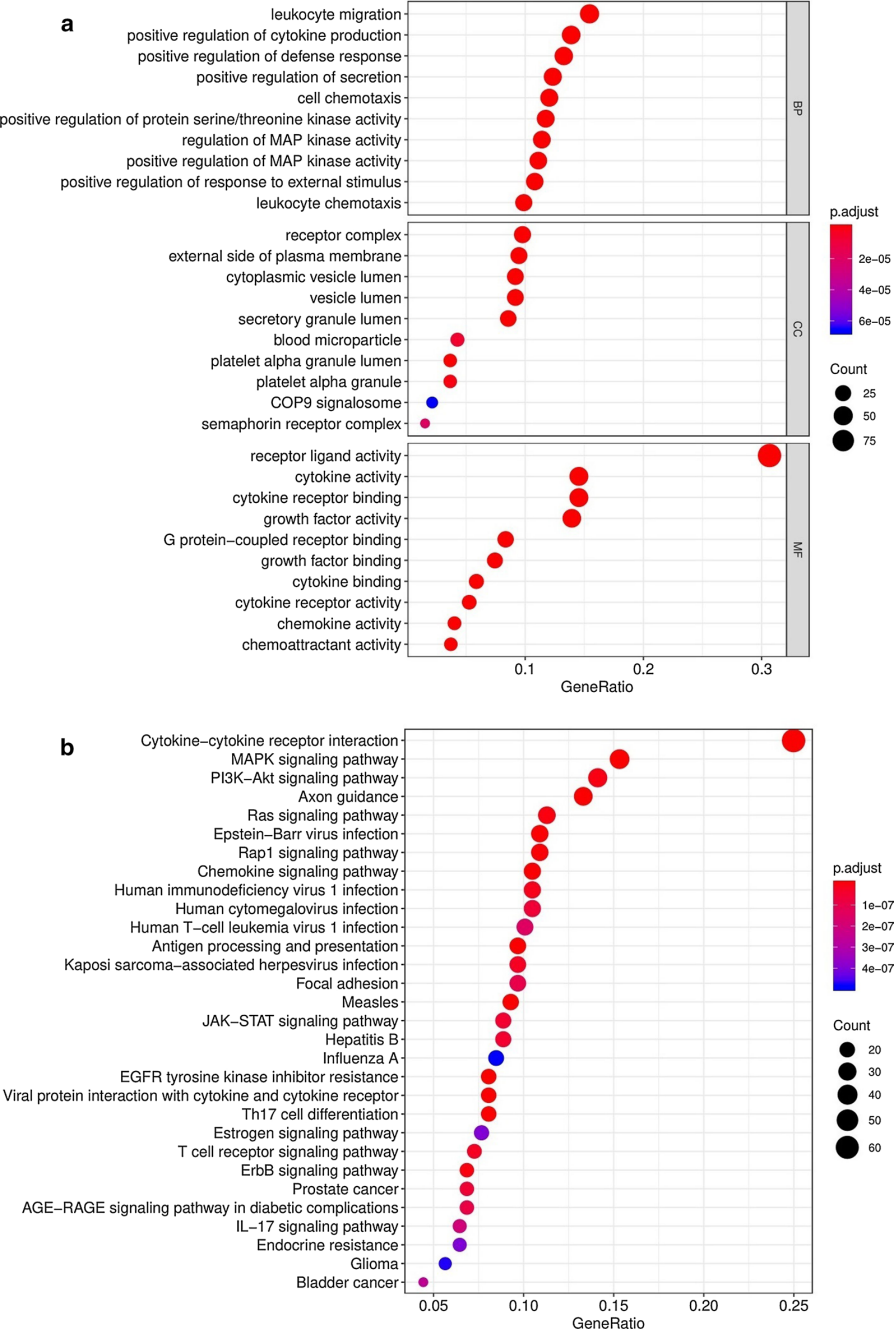
Functional enrichment analysis of differentially expressed immune-related genes. **a** Gene ontology analysis; From top to bottom, the figure represents biological process, cellular component and molecular function, respectively. **b** The top 30 most significant Kyoto Encyclopedia of Genes and Genomes (KEGG) pathways

A total of 329 immune‐related genes (267 upregulated and 62 downregulated) and 117 TFs (108 upregulated and 9 downregulated) were identified as differentially expressed in HCC tissues compared with normal tissues. The heat maps revealed that HCC samples can be obviously distinguished from the normal samples according to differentially expressed immune‐related genes and TFs (Fig. 1a, b). Volcano plots shows the distribution of differentially expressed immune‐related genes and TFs between HCC and normal controls (Fig. 1c, d). The 329 differentially expressed immune‐related genes were further analyzed by GO and KEGG analysis. GO analysis revealed that primary functional categories in the biological processes (BP) were leukocyte migration, positive regulation of cytokine production and positive regulation of defense response. For cellular components (CC), the major enriched GO terms were receptor complex and external side of plasma membrane. The most enriched cellular components (CC) were receptor ligand activity, cytokine activity and cytokine receptor binding (Fig. 2a). KEGG pathway indicated that the differentially expressed immune‐related genes were mainly involved in Cytokine–cytokine receptor interaction, MAPK signaling pathway and PI3K-Akt signaling pathway (Fig. 2b).

### Screening of immune-related genes with prognostic value in HCC

**Fig. 3:**
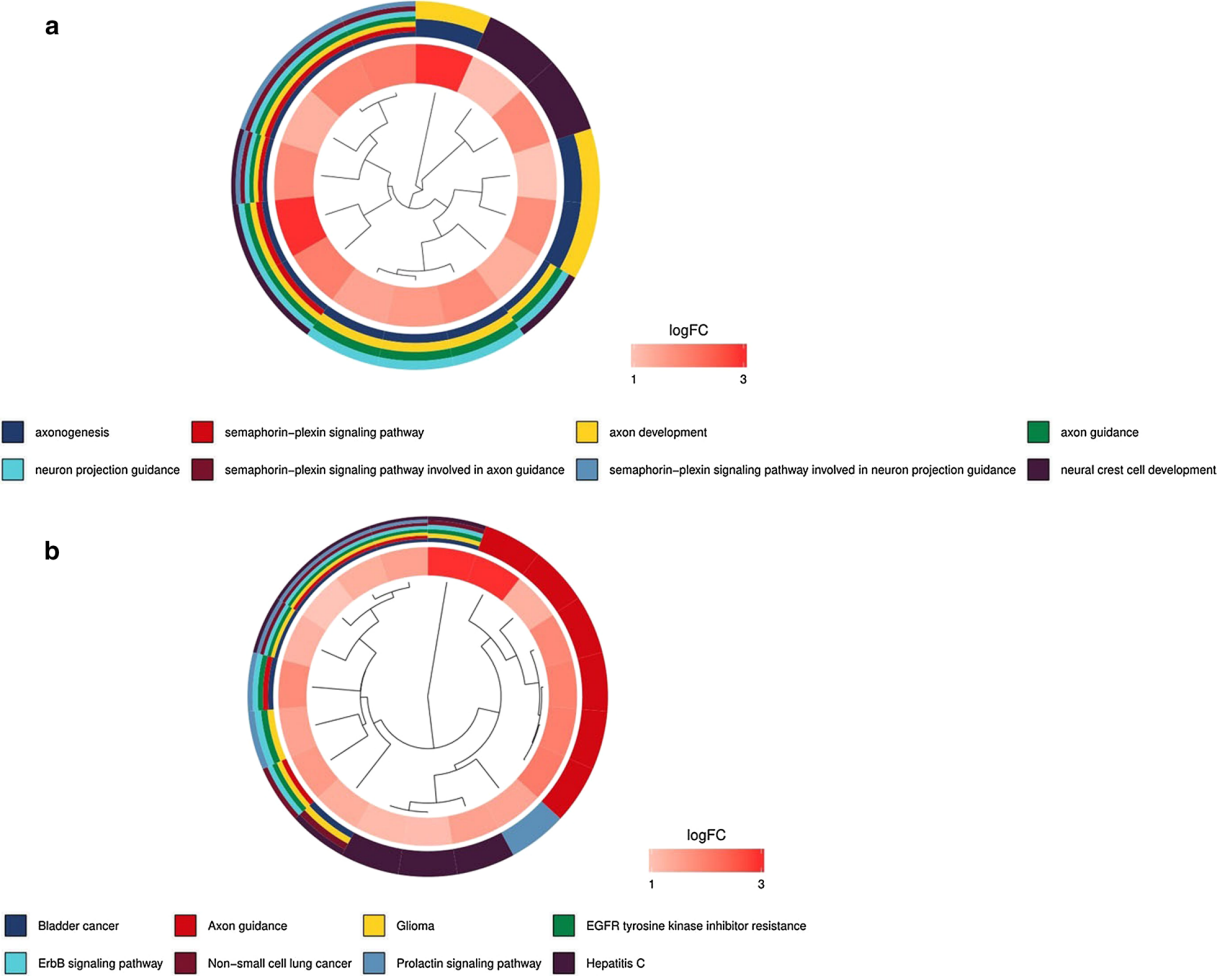
Functional enrichment analysis of prognostic immune‐related genes. **a** Gene ontology analysis; The outer circle is a bar plot where the height of the bar indicates the significance of GO terms. The inner ring shows a scatter plot of the expression (logFC) of differentially expressed immune-related genes in each enriched gene ontology term. **b** Top 8 enriched KEGG pathways for the prognostic immune-related genes

To determine the differentially expressed immune‐related genes with prognostic characteristics, the 329 genes expression in the 337 HCC samples were evaluated by univariate Cox analysis. Totally, 64 immune‐related genes were found to be related to OS. The prognostic immune‐related genes were shown in Table 1. GO and KEGG analysis suggested that these prognostic immune‐related genes mainly participated in semaphorin–plexin signaling pathway, ErbB signaling pathway, Hepatitis C. These pathways were shown to be significantly correlated with the development of cancer (Fig. 3a, b).

**Table 1:** General characteristics of prognostic immune‐related genes

Gene symbol	logFC	FDR	HR	P-value
ANGPT1	1.547874506	6.91E−08	1.590922357	0.000591915
AP3B1	1.258960075	4.13E−23	1.151791139	0.000260954
BIRC5	4.814625294	4.67E−26	1.029946214	6.85E−06
BRD8	1.33190695	2.91E−23	1.126741791	0.001638443
CACYBP	1.748309409	3.02E−25	1.050633887	3.28E−07
CANX	1.102288423	1.49E−20	1.004139804	0.002069851
CD320	1.398696619	7.68E−20	1.020939754	0.000868482
CDK4	1.382141701	4.03E−18	1.036790715	4.85E−06
CKLF	1.626423097	7.18E−22	1.04078873	0.007319747
CMTM3	1.27678529	2.27E−07	1.038755913	0.007999383
CSPG5	3.246433614	1.01E−20	1.442576573	0.002352963
DCK	1.344974898	4.37E−15	1.126006789	2.12E−05
EDNRA	1.11092361	0.000755102	1.20178986	0.007367814
EED	1.255516772	1.86E−23	1.273523516	0.006813691
EGF	5.593952491	1.61E−05	1.364265199	0.003925913
FABP6	5.416594713	6.99E−06	1.077060148	0.003829421
FIGNL2	2.311354994	2.27E−10	1.531957465	0.003674266
GMFB	1.023249329	5.89E−16	1.115051082	0.000394597
GRN	1.20949936	6.40E−20	1.003274994	0.002862808
HDAC1	1.026001495	3.30E−18	1.034280566	6.51E−06
HRAS	1.585403435	1.36E−23	1.03121775	0.000418996
HSP90AA1	1.057758216	3.20E−18	1.003949782	0.000797033
HSPA4	1.230240905	1.44E−24	1.043748238	4.02E−07
IFI30	1.12334786	2.02E−08	2.049042625	0.000288336
IL17D	3.841741735	2.39E−12	1.078068488	0.002795608
IRF5	1.245306971	1.70E−16	1.139673698	0.007186085
ISG20L2	1.25060274	2.91E−23	1.105907508	1.49E−05
KITLG	1.899602508	7.45E−12	1.230984408	1.18E−05
MAP2K2	1.318560825	1.04E−24	1.018137718	0.000185682
MAPK3	1.378505311	1.65E−24	1.071629584	8.76E−05
MAPT	3.72836937	6.84E−24	1.462317341	2.13E−05
MAVS	1.54893751	2.05E−23	1.095286557	0.000752941
MDK	4.344790262	1.73E−23	1.001931972	0.006701426
MICB	1.915426385	3.61E−15	1.142650746	0.004845855
NDRG1	1.903124723	1.66E−11	1.010625267	2.24E−10
NR6A1	1.979350372	1.66E−21	1.276712088	5.01E−05
NRAS	1.044758631	2.90E−16	1.061372436	5.25E−07
OSGIN1	1.503239816	2.23E−07	1.003746458	0.005488354
PGF	1.843288391	9.59E−13	1.15659945	0.008495924
PLCG1	1.664062936	1.34E−20	1.104101656	0.001844618
PLXNA1	2.023359471	6.32E−17	1.163576469	1.21E−05
PLXNA2	1.33437797	9.02E−20	1.217021605	0.008639913
PLXNA3	2.123267013	1.14E−14	1.171143858	0.001892787
PPARG	1.20822674	1.43E−10	1.075684273	0.000601202
PPIA	1.30098489	9.41E−26	1.011506615	2.02E−05
PSMD10	1.361273275	1.60E−24	1.044232757	0.000519359
PSMD14	1.205250947	3.10E−23	1.097579688	9.44E−08
PSMD2	1.147131985	1.89E−23	1.019634542	7.89E−05
PSME3	1.169085013	9.38E−23	1.048566596	3.71E−05
RBP2	4.984713661	5.87E−05	1.018064173	0.000257117
S100A10	1.91428128	8.62E−18	1.002461873	0.003233598
S100A11	1.70750925	3.49E−05	1.001204132	0.001558109
S100A6	1.84072664	9.98E−05	1.00128807	0.006992081
SEMA3F	1.920566843	1.66E−26	1.087890549	0.009965887
SEMA4F	2.078275883	5.47E−13	1.355626695	0.008065557
SEMA5B	3.054072412	2.36E−25	1.170460784	0.009801081
SHC1	1.541730574	1.57E−24	1.011068693	0.001450078
SKIV2L	1.150515308	6.64E−24	1.052992847	0.008204257
SORT1	1.846919333	1.23E−16	1.042842316	0.009849761
SRC	1.862441994	4.49E−10	1.044117871	0.004570868
STC2	2.825099592	5.37E−18	1.030706624	0.000996678
TNFRSF11A	1.526321785	0.00039172	1.317452099	0.001542218
TRAF3	1.193726485	2.95E−15	1.252886114	0.000628119
ZYX	1.078749242	2.23E−12	1.010389289	0.002188303

### TF regulatory network

**Fig. 4:**
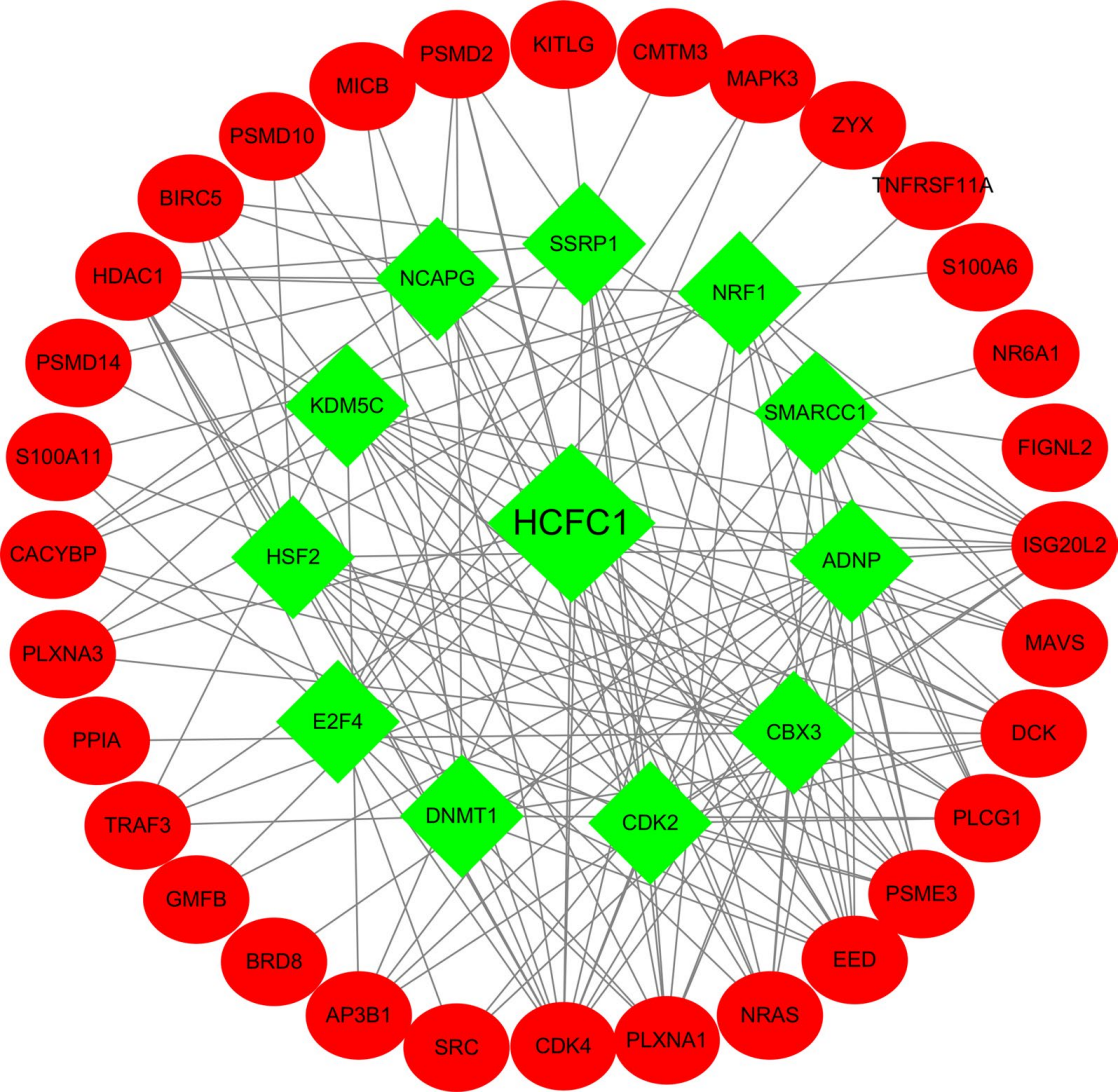
The main regulatory network constructed based on prognosis-related transcription factors and prognostic immune‐related genes. The red circular represent differentially expressed prognostic immune‐related genes and the green diamond represent prognosis-related transcription factors, respectively

To explore the regulatory mechanisms of prognostic immune‐related genes, we selected the prognosis-related TFs among HCC patients. Univariate Cox regression analysis detected that 54 TFs were correlated to patient overall survival (Table 2). We established a regulatory network according to 54 TFs and 64 immune‐related genes. We built the TF regulatory network in three steps: (1) 54 prognosis-related TFs and 64 prognostic immune‐related genes were selected. (2) The correlation test between each TF and each immune‐related gene was conducted using cor.test function, whose core method was Pearson test. Based on the cut-off criteria, 12 prognosis-related TFs and 32 prognostic immune‐related genes were identified to establish the network. (3) Here, we utilized Cytoscape to construct and visualize the main regulatory network. As shown in Fig. 4, HCFC1 regulated most of the immune‐related genes and occupied the dominant position. This transcriptional regulatory network revealed the regulatory relationships among these immune-related genes.

**Table 2:** General characteristics of prognostic TFs

Transcription factors	logFC	FDR	HR	P-value
ADNP	1.111342546	9.27E−19	1.058451885	0.002474062
ARID3A	3.256724811	1.13E−17	1.045574467	0.008276291
BATF	1.543612769	0.000474464	1.019593757	0.002497978
BRCA1	1.502983199	1.38E−11	1.29448955	0.002745177
CBX2	3.45997956	3.30E−23	1.186383883	8.38E−07
CBX3	1.221810833	4.97E−24	1.02933704	7.80E−05
CBX8	1.871864633	2.01E−25	1.198292974	0.000174928
CDK2	1.065195274	2.01E−11	1.095803202	4.91E−05
CDK7	1.195768424	8.52E−24	1.068973096	0.006247221
CENPA	4.889402591	1.35E−25	1.179109237	1.69E−09
DNMT1	1.906233242	5.00E−20	1.063445055	0.000751632
DNMT3A	1.996223959	1.03E−22	1.191390711	0.00019982
E2F3	1.418711623	6.75E−13	1.103109241	0.009036249
E2F4	1.206904224	1.21E−21	1.071429425	7.56E−05
E2F7	4.285471393	3.88E−23	1.493962132	0.003027944
EED	1.255516772	1.86E−23	1.273523516	0.006813691
EHMT2	2.012149818	5.91E−26	1.053983491	0.003998918
EP400	1.153527493	6.14E−18	1.389348084	0.000263802
EZH2	3.104108848	6.03E−26	1.174112818	2.35E−07
FOXK1	1.929438524	1.31E−23	1.273342188	8.24E−05
FOXM1	4.385308416	2.64E−25	1.063687255	9.77E−06
H2AFX	2.17270105	1.08E−22	1.020169766	5.70E−05
HCFC1	1.374622669	6.19E−23	1.098505344	5.22E−05
HDAC1	1.026001495	3.30E−18	1.034280566	6.51E−06
HSF2	1.292877962	1.21E−17	1.201940551	0.000270883
IRF5	1.245306971	1.70E−16	1.139673698	0.007186085
JMJD6	1.104572283	1.62E−16	1.147919917	5.09E−05
KDM1A	1.000926229	4.91E−21	1.069745907	1.04E−05
KDM5C	1.001989724	8.01E−15	1.061530094	0.004103255
LEF1	3.56711144	1.37E−16	1.117405004	0.009987013
LMNB1	2.014673486	1.11E−14	1.051211802	2.84E−05
MYBL2	5.305148325	2.29E−25	1.026913373	1.05E−06
NCAPG	4.548929596	8.18E−26	1.16392489	1.36E−08
NRF1	1.019899435	3.23E−21	1.272301351	0.002963181
POLR3A	1.047070233	7.33E−22	1.317574436	0.000100998
POLR3G	1.184477344	1.04E−12	1.99297179	4.39E−05
POU2F1	1.093590466	3.01E−18	1.735254091	0.000533304
PPARG	1.20822674	1.43E−10	1.075684273	0.000601202
PRKDC	1.5144719	1.73E−19	1.066891069	0.000167653
RBP2	4.984713661	5.87E−05	1.018064173	0.000257117
SAP30	1.292845644	4.27E−17	1.149649393	9.86E−07
SCML2	2.073651192	7.01E−18	1.293235072	0.001143596
SIRT6	1.273792186	1.95E−22	1.100603266	0.000366707
SMAD2	1.054220332	4.40E−21	1.551982434	0.001049848
SMARCA4	1.640491763	2.66E−25	1.048414219	0.006425207
SMARCB1	1.142844803	6.87E−21	1.019578446	0.003634163
SMARCC1	1.173821643	1.48E−18	1.066416216	0.00098243
SNAPC2	1.16836701	1.83E−22	1.13661385	1.34E−05
SOX4	2.015579447	5.09E−07	1.01963735	0.007903766
SRC	1.862441994	4.49E−10	1.044117871	0.004570868
SSRP1	1.014534161	2.39E−20	1.038495674	2.58E−05
SUMO2	1.02926143	8.48E−22	1.022123756	0.003732059
TRIM28	1.405145621	7.40E−24	1.006131502	0.001669502
ZBTB17	1.051057022	9.98E−23	1.240907981	6.11E−06

### Construction of immune-related prognostic model for HCC

**Fig. 5:**
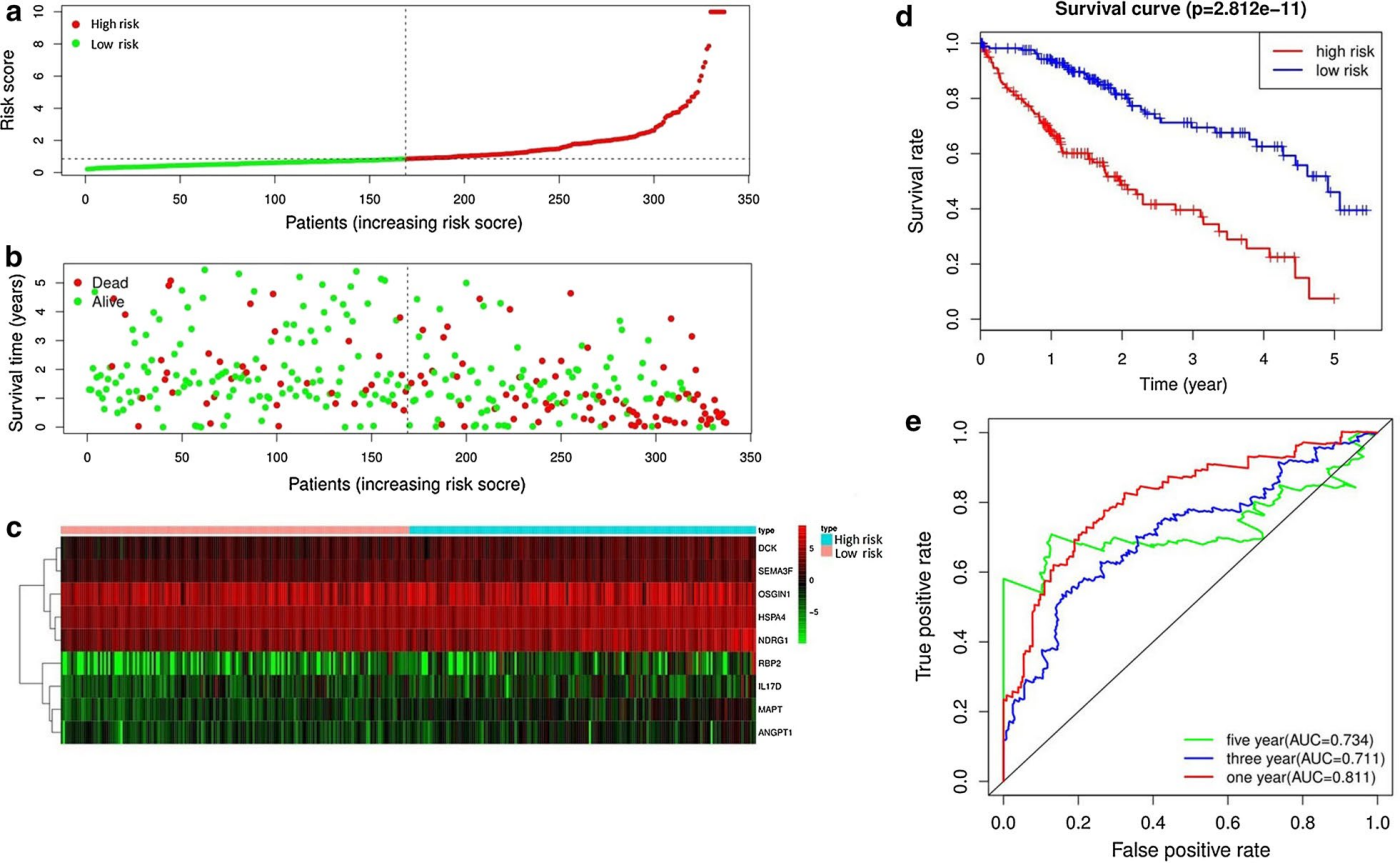
Construction of an immune-related prognostic signature for hepatocellular carcinoma. **a** The risk score distribution of HCC patients in the The Cancer Genome Atlas (TCGA) database. **b** Survival status and duration of patients. **c** Heatmap of the nine immune‐related genes expression in HCC patients. **d** Survival curves for the low risk and high risk groups. **e** Receiver operating characteristic curve (ROC) analysis predicted overall survival using the risk score

**Fig. 6:**
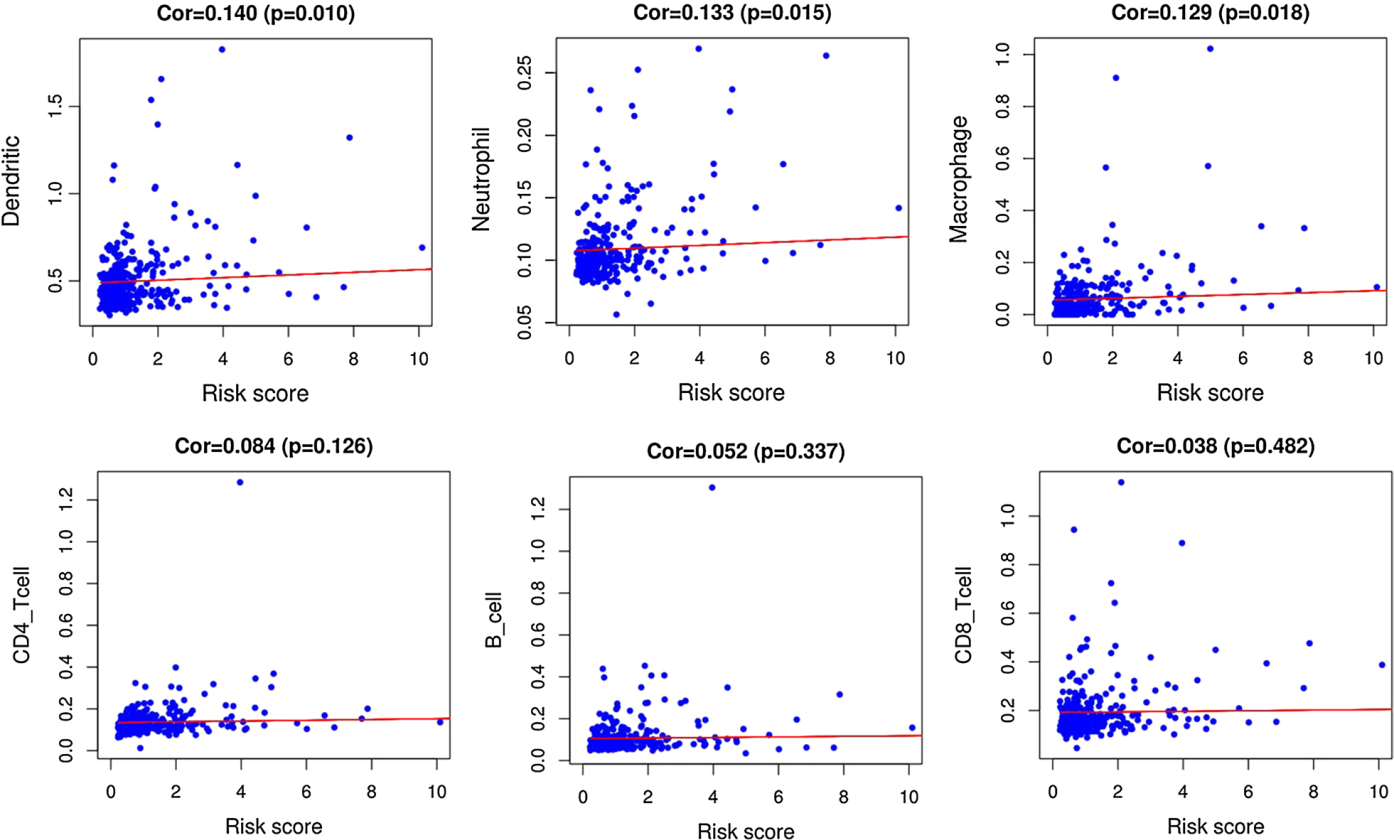
Relationships between the risk score model and infiltration abundances of six types of immune cells

64 prognostic immune‐related genes were subjected to Lasso Cox analysis and 21 genes were filtered out. Then multivariate Cox analysis were performed and nine genes were finally selected to establish a prognostic model. The formula was shown as: risk score =  (0.2940 * expression level of ANGPT1) +  (0.1753 * expression level of MAPT) +  (0.1066 * expression level of DCK) +  (0.0706 * expression level of SEMA3F) +  (0.0703 * expression level of IL17D) +  (0.0311* expression level of HSPA4) +  (0.0204 * expression level of RBP2) +  (0.0084 * expression level of NDRG1) +  (0.0052 * expression level of OSGIN1). All the nine genes were risky prognostic genes with hazard ratio > 1. Risk scores were based on genes expression levels multiplied its corresponding regression coefficients. Regression coefficients were calculated by multivariate Cox regression. The risk scores were not only related to the expression levels of these genes, but also related to the correlation coefficients. Then 337 HCC samples were classified into a high risk group (n = 168) and low risk group (n = 169) based on the median risk score (Fig. 5a). The survival overview and gene expression heatmap were presented in Fig. 5b–c. Survival analysis indicated that patients in the high risk group showed markedly poorer overall survival than those in the low risk group (P < 0.0001; Fig. 5d). The area under the ROC curve for 1 year, 3 year, and 5 year OS were 0.811, 0.711, 0.734, suggesting that this prognostic model exhibited a good sensitivity and specificity (Fig. 5e). The relationships between the risk score model and immune cell infiltration were investigated. As shown in Fig. 6, dendritic cells, neutrophil and macrophage were positive correlated with risk score. However, no significant correlations were observed between B cells, CD4+ T cells, CD8+ T cells and risk score.

### Independence of immune-related prognostic signature from other clinical factors

207 HCC patients with clinical information containing gender, age, histological grade, pathologic stage and TNM stage were selected for further analysis. Univariate and multivariate Cox regression analyses were conducted to assess the independent predictive power of immune-related prognostic signature. Univariate analysis indicated that pathological stage (P < 0.0001), T classification (P < 0.0001) and immune-related prognostic model (P < 0.0001) were markedly correlated with overall survival. After the multivariate analysis, only immune-related prognostic model remained as an independent prognostic factor associated with OS (P < 0.0001; Table 3).

**Table 3:** Univariate and multivariate analyses of overall survival in hepatocellular carcinoma patients of TCGA

Variables	Univariate analysis		Multivariate analysis	
	Hazard ratio (95% CI)	P-value	Hazard ratio (95% CI)	P-value
Age	1.007 (0.988–1.026)	0.495	1.010 (0.990–1.030)	0.322
Gender	0.755 (0.461–1.237)	0.264	1.168 (0.668–2.044)	0.586
Histologic grade	0.915 (0.660–1.269)	0.596	0.888 (0.624–1.264)	0.510
Pathologic stage	1.782 (1.388–2.288)	5.85E−06	0.893 (0.328–2.428)	0.825
T classification	1.725 (1.370–2.172)	3.50E−06	1.784 (0.732–4.348)	0.203
M classification	3.141 (0.984–10.021)	0.053	1.505 (0.387–5.852)	0.555
N classification	1.604 (0.391–6.576)	0.512	2.267 (0.353–14.556)	0.388
Prognostic model	1. 126 (1.089–1.165)	5.02E−12	1.120 (1.079–1.162)	1.88E−09

### Genetic alterations of nine immune-related prognostic genes

**Fig. 7:**
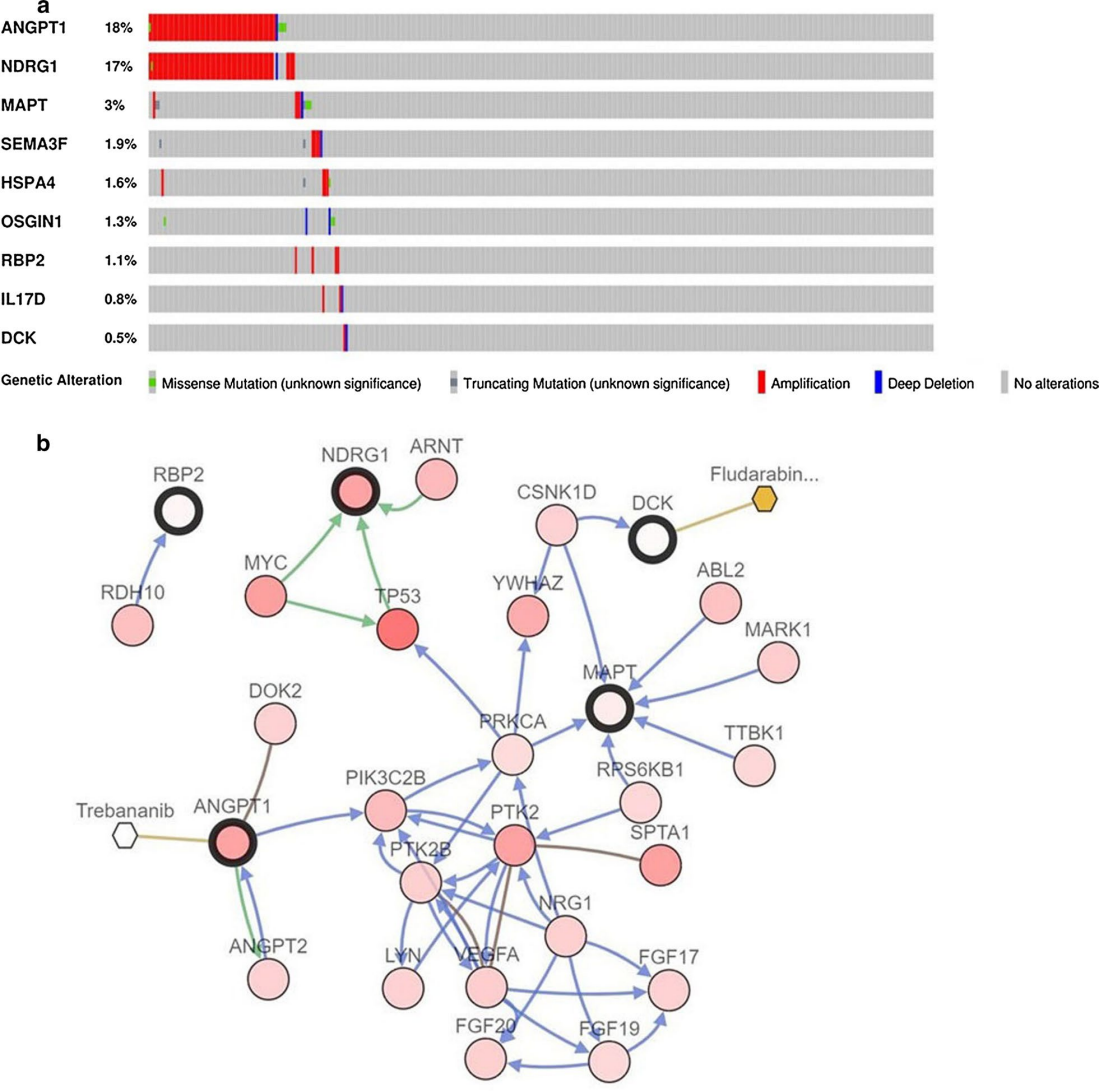
Genetic alterations and biological function of nine prognostic immune‐related genes. **a** The genetic alteration of nine genes in HCC patients using the cBioPortal database. **b** The network contained 59 nodes, including nine query genes and the 50 most frequently altered neighbor genes (only five out of nine were correlated with the 50 genes). The relationship between key prognostic immune‐related and cancer drugs was illustrated

The cBioPortal tool was employed to analyze genomic alternations and potential drugs of nine immune-related prognostic genes. As shown in Fig. 7a, ANGPT1 and NDRG1 were most commonly altered genes. Amplification was the main frequent genetic alterations and the nine immune‐related prognostic genes altered in 94 (25.2%) of 373 cases. Figure 7b illustrated the network built by nine immune-related prognostic genes and their 50 most frequently mutated neighbor genes. Anticancer drugs targeting these genes were exhibited. Among them, two genes (DCK and ANGPT1) were currently regarded as drugs targets. We considered that other genes might act as potential novel therapeutic targets.

### Validation of the immune-related prognostic signature by ICGC database

**Fig. 8:**
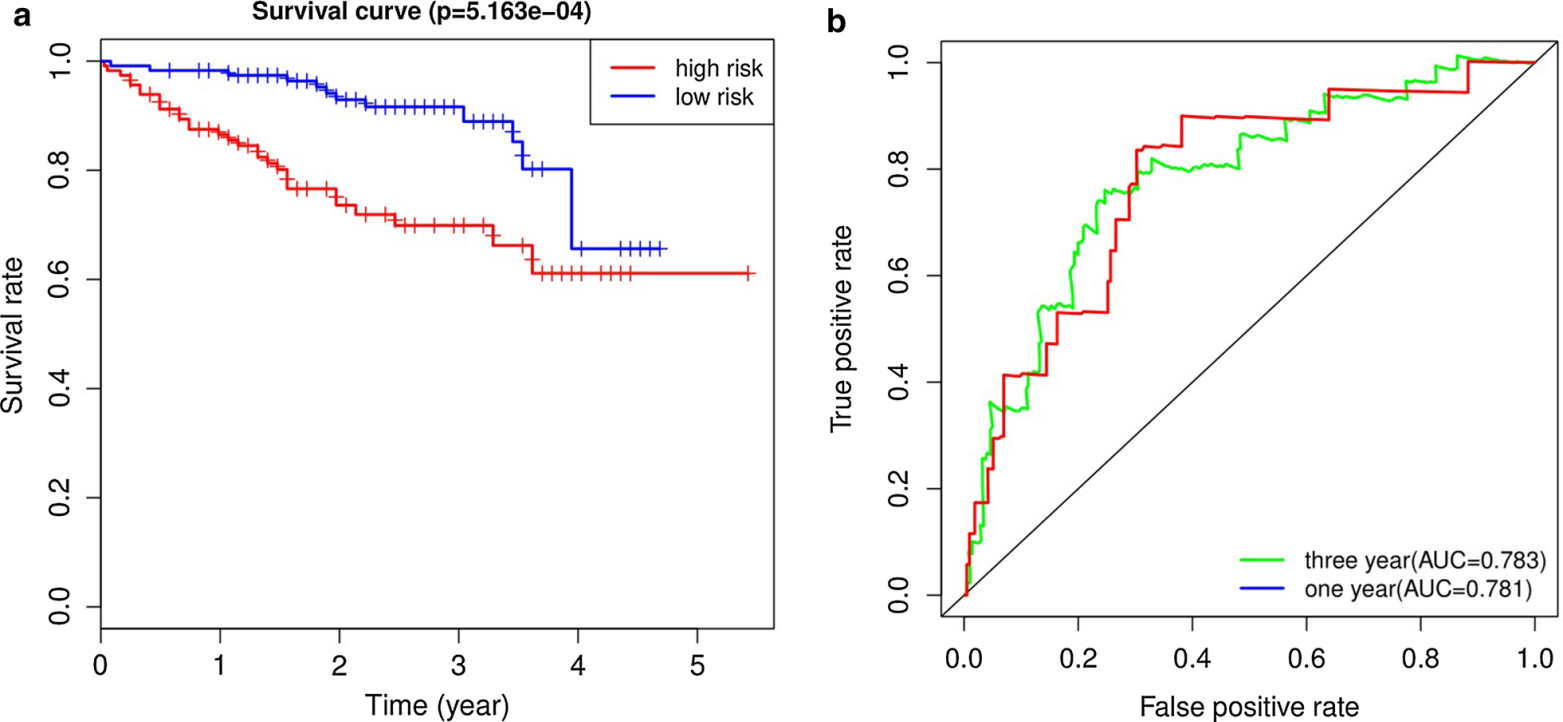
ROC and Kaplan–Meier analysis of the nine-gene signature in International Cancer Genome Consortium (ICGC) datase. **a** The Kaplan–Meier curve of the overall survival between the high risk and low risk groups stratified by the median risk score in ICGC. **b** ROC analysis of the predictive efficiency of the nine-gene prognostic model on overall survival based on risk score

The ICGC database including 231 HCC samples were used for the validation of the immune-related signature. According to the median risk score, we divided patients into high risk (n = 115) and low risk groups (n = 116). In agreement with results of TCGA cohort, the Kaplan–Meier curve demonstrated that patients in the high risk group exhibited markedly poorer overall survival than those in the low risk group (P < 0.001; Fig. 8a). The AUCs for 1 year and 3 year OS were 0.781 and 0.783, demonstrating good performance of the immune-related signature in predicting OS (Fig. 8b). Because there was only one patient with a 5-year follow-up period, we did not plot a 5-year ROC curve.

## Discussion

Hepatocellular carcinoma still remains a lethal malignancy with extremely unfavorable prognosis globally. Precise prediction of HCC overall survival is of great significance for the choice of therapeutic methods and amelioration of prognosis. To date, there are no reliable and effective biomarkers to accurately predict the survival of HCC patients. There is a critical demand to identify robust biomarkers and prediction model to forecast HCC outcomes.

In the current study, based on the analysis of TCGA dataset, 329 differentially expressed immune‐related genes were screened out from 374 cases of HCC and 50 normal tissues. According to the results of the GO enrichment, the mentioned genes were primarily associated with immune response. The KEGG pathways were mostly concentrated on several cancer-related pathways (e.g., MAPK signaling pathway and PI3K–Akt signaling pathway). In univariate regression analysis on the differentially expressed immune‐related genes, 64 genes were detected to display significant association with OS. To delve into the regulatory mechanisms of the prognostic immune‐related genes, a TF-mediated network was built to reveal crucial TFs that are capable of regulating these immune‐related genes. The main network suggested that HCFC1 was the critical key regulator in the network. HCFC1 (host cell factor C1), belongs to the host cell factor family. It is noteworthy that a recent study reported HCFC1 as a clinically hub gene that was remarkably correlated with the survival time, grade and TNM stage of HCC patients [31]. To date, the contribution of HCFC1 to the development of HCC remains unclear. Further experimental evidence is needed to explore the molecular mechanisms of HCFC1 in HCC.

Recently, gene signatures according to aberrant mRNA have attracted wide attention and revealed the huge potential in prognosis prediction of HCC. For instance, Long et al. built a four-gene prognostic model that showed a good performance for HCC prognosis prediction [32]. Another study also constructed a six-gene prognostic signature for HCC overall survival prediction based on gene expression data from TCGA [33]. A recent study investigating the prognostic value of TP53-associated immune genes in HCC identified and validated a two-gene (TREM1 and EXO1) prognostic model [34]. However, these studies did not use a large number of samples to comprehensively explore the relationship between immune genes and prognosis of HCC. Compared with the previous researches, this study has several advantages: (1) we utilized the specialized immunology database, which allowed us to analyze as many immune genes as we can. To our knowledge, this is the first study to explore the relationships between a large number of immune-related genes and prognosis in HCC patients. (2) We obtained a number of prognostic immune-related genes and established a novel immune-related prognostic model. This prognostic model exhibited a prominent performance for OS prediction based on TCGA database. According to the in-depth analysis, the immune-related prognostic model was demonstrated to be an independent prognostic indicator after adjusting to other clinical factors. Subsequently, such model that consists of nine immune‐related genes was then successfully validated as a prognostic factor in an independent ICGC dataset. All the mentioned results revealed that immune-related prognostic model could act as an effective marker for HCC prognosis prediction.

To characterize the tumor immune microenvironment status, the relationships between immune-related prognostic model and immune cell infiltration were investigated. The data here indicated that higher infiltration levels of dendritic cells, neutrophil and macrophage may be observed in high risk patients. Dendritic cells, neutrophil and macrophage displayed positive correlation with immune-related prognostic model, revealing that the model may serve as predictor for increased immune cells infiltration. A recent study reported that intratumoral infiltration by dendritic cells had a close relation to the poor prognosis in HCC patients [35], which is consistent with our findings. An existing study reported that neutrophil infiltration within HCC might display an association with a poor clinical outcome [36]. Neutrophils contribute to the activation, regulation and effector function of immune cells [37]; they are also capable of HCC progression by secreting a wide range of cytokines [38], thereby demonstrating their crucial role in the pathogenesis of HCC. A number of studies have reported that increased macrophages were related to poor prognosis in HCC [39]. Macrophages infiltration within the tumor microenvironment could facilitate tumor growth, angiogenesis, invasion, as well as metastasis [40]. Targeting macrophages have been considered a promising adjuvant immunotherapy for HCC patients [41, 42]. The role of immune cells in HCC has not been fully elucidated. It may be a promising way to cure HCC by broadening the relationship between immune cells and tumor progression.

Nine immune-related genes that constituted the prognostic model were identified as potential biomarkers in HCC. Out of the nine genes, RBP2, NDRG1 and HSPA have been well studied in HCC compared to other immune-related genes. RBP2 (retinol binding protein 2), belongs to the Fatty-acid binding protein (FABP) family, was reported to be involved in the pathogenesis of diverse types of cancer [43]. It has been previously evidenced that RBP2 was overexpressed in HCC and was associated with unfavorable prognosis of HCC [44]. Overexpressed RBP2 was markedly correlated with AFP and TNM stage. RBP2 might be critical to the angiogenesis and progression of HCC. Elevated NDRG1 expression was observed in HCC and dramatically related to overall survival and tumor stage [45]. NDRG1 was suggested to play significant roles in the metastasis, recurrence and and prognosis of HCC [46]. Moreover, overexpressed NDRG1 displayed a significant association with hepatocarcinogenesis [47]. Accordingly, targeting NDRG1 might act as an attractive therapeutic strategy for HCC. HSPA4, also known as hsp70, was demonstrated to enhance the proliferation, invasion and metastasis of various cancers [48]. High Expression of HSPA4 was significantly correlated with worse overall survival of HCC; it was aslo an independent prognostic parameter for OS [49]. Given the findings here, HSPA4 demonstrated huge potential as a therapeutic target in HCC treatment. In the HCC tissues, up-regulated expression of ANGPT1 was detected as compared with normal liver tissues [50], whereas the prognostic implication in HCC was not studied. A previously study showed IL17D had a diagnostic value for HCC and the DNA methylation status of IL17D was related to OS [51]. Nevertheless, the specific role of IL17D in HCC has been rarely known. Only one study has reported OSGIN1 may be a tumor suppressor that was downregulated in HCC, which contradicted our findings [52]. Its exact role in HCC is as yet unclear. Thus far, no relevant research reported MAPT, DCK and SEMA3F in HCC. Further researches are required to elucidate the function of these potential immune-related genes in HCC.

Some shortcomings of this study should be addressed. First, this study was completely based on public databases and the results should be external validated by further experiments. Second, the efficiency of the immune-related prognostic model should be identified in a large number of HCC samples using experimental methods. Third, the biological functions of nine immune-related genes in HCC require further examined by a series of experiments.

## Conclusion

In conclusion, for the first time, numerous immune-related genes were detected to be significantly related to HCC prognosis by comprehensive analyses. Moreover, we constructed a novel immune-related prognostic model as an independent prognostic predictor for HCC. Validation in an external ICGC database further confirmed the prognostic value of this model. This prognostic model may also serve as predictor for increased immune cells infiltration, proving its key role in tumor immune microenvironment. The current study deepens our understanding of immune-related genes in HCC and provides new potential prognostic and therapeutic biomarkers.

## Data Availability

Not applicable.

## Abbreviations

HCC: Hepatocellular carcinoma
TCGA: The Cancer Genome Atlas
DEGs: Differentially expressed genes
TFs: Transcription factors
PD-1: Programmed cell death-1
ICGC: International Cancer Genome Consortium
TIMER: Tumor immune estimation resource
GO: Gene ontology database
KEGG: The Kyoto encyclopedia of genes and genomes
ROC: Receiver operating characteristic
AUC: Area under the ROC curve
OS: Overall survival
